# Cinnamon-Derived Hierarchically Porous Carbon as an Effective Lithium Polysulfide Reservoir in Lithium–Sulfur Batteries

**DOI:** 10.3390/nano10061220

**Published:** 2020-06-22

**Authors:** Ranjith Thangavel, Aravindaraj G. Kannan, Rubha Ponraj, Karthikeyan Kaliyappan, Won-Sub Yoon, Dong-Won Kim, Yun-Sung Lee

**Affiliations:** 1 School of Chemical Engineering, Chonnam National University, Gwang-ju, 61186, Korea; ranjith.cecri@gmail.com; 2 Department of Energy Science, Sungkyunkwan University, Suwon, 16419, Korea; wsyoon@skku.edu; 3 Department of Chemical Engineering, Hanyang University, Seoul, 04763, Korea; aravindgk@gmail.com (A.G.K.); rubhap@gmail.com (R.P.); 4 Department of Chemical Engineering, University of Waterloo, Waterloo, ON N2L 3G1, Canada; karthik506@gmail.com

**Keywords:** lithium-sulfur batteries, bio-mass carbon, hierarchical nanostructures, polysulfides

## Abstract

Lithium–sulfur batteries are attractive candidates for next generation high energy applications, but more research works are needed to overcome their current challenges, namely: (a) the poor electronic conductivity of sulfur, and (b) the dissolution and migration of long-chain polysulfides. Inspired by eco-friendly and bio-derived materials, we synthesized highly porous carbon from cinnamon sticks. The bio-carbon had an ultra-high surface area and large pore volume, which serves the dual functions of making sulfur particles highly conductive and acting as a polysulfide reservoir. Sulfur was predominantly impregnated into pores of the carbon, and the inter-connected hierarchical pore structure facilitated a faster ionic transport. The strong carbon framework maintained structural integrity upon volume expansion, and the unoccupied pores served as polysulfide trapping sites, thereby retaining the polysulfide within the cathode and preventing sulfur loss. These mechanisms contributed to the superior performance of the lithium-sulfur cell, which delivered a discharge capacity of 1020 mAh g^−1^ at a 0.2C rate. Furthermore, the cell exhibited improved kinetics, with an excellent cycling stability for 150 cycles with a very low capacity decay of 0.10% per cycle. This strategy of combining all types of pores (micro, meso and macro) with a high pore volume and ultra-high surface area had a synergistic effect on improving the performance of the sulfur cathode.

## 1. Introduction

The ever increasing energy demands for portable electronic devices and electric vehicles, along with the demands of grid-scale energy storage devices for the back-up and load-leveling of intermittent renewable energy sources such as solar cells and wind mills, have shifted the focus of battery research to high energy density storage devices such as lithium–sulfur (Li–S) batteries [1,2]. The high theoretical specific capacity (1672 mAh g^−1^), along with the abundant availability of sulfur and its environmental friendliness, make Li–S batteries attractive candidates for these applications [3,4]. However, the inherent disadvantages of these batteries, including (a) the highly insulating nature (both electronically and ionically) of sulfur and its discharge products, (b) the dissolution of intermediate lithium polysulfides (Li_2_S*_n_*, where 4 < *n <* 8) in the electrolyte, (c) the migration and poisoning of lithium metal anodes by lithium polysulfides and (d) the volume expansion of the sulfur cathode upon lithiation, result in a low coulombic efficiency, poor sulfur utilization and poor cycle life, which renders them unsuitable for practical applications in the current state [5,6,7].

To address these issues, the sulfur cathode design has been reconfigured by embedding sulfur into highly conductive porous carbons [8,9,10], encapsulating sulfur using conductive polymer coatings [11,12,13], utilizing Li_2_S as an initial active material [14] and utilizing lithium polysulfide adsorbents to trap the lithium polysulfides within the cathode to enhance the cycling performance of Li–S cells [15,16,17]. In addition, strategies such as protecting the lithium metal surface [18,19,20], utilizing non-lithium metal anodes [21], modifying the electrolyte composition through a rational selection of solvents and electrolyte additives [22,23] and modifying the separator to block the migration of lithium polysulfides to the anode side have been adopted to mitigate the capacity-fading observed upon cycling [24,25,26]. Among these approaches, different types of porous carbon have been successfully employed in sulfur cathodes to enhance the electronic conductivity, accommodate sulfur and act as a reservoir for the soluble polysulfides [25,27]. Conventional porous carbons can be obtained from the activation of fossil fuels such as coke and coal, which are environmentally harmful and unsustainable. Carbons derived from various bio- and eco-friendly resources such as coffee waste, pine cones and soy bean residue have been widely used in lithium-ion batteries, capacitors, catalysis, separation and adsorption processes [28,29,30]. Recently, porous carbons derived from sustainable resources have become potentially interesting for Li–S batteries. Porous carbons derived from bio-mass precursors such as chitosan, coffee waste grounds, peanut shell and rice husk have been previously studied for Li–S batteries [31,32,33]. However, most of the research limits the sulfur content to 50 wt.% along with low mass loading conditions. A large amount of sulfur content (70 wt.%) in porous carbon is very essential in order to achieve high energy density Li–S batteries. The low surface area and low pore volume in bio-mass derived porous carbon limits the sulfur content inside the porous carbon. A porous carbon with a large surface and pore volume can accommodate a large amount of sulfur active material. The porous carbon with tailored micropores/mesopores can prevent sulfur loss by immobilizing the sulfur, while mesopores provide rapid lithium ion transport and macropores absorb the electrolyte [34,35]. A bio-mass carbon with a combination of a high surface, high pore volume and hierarchical porous structure can greatly boost the performance of Li–S batteries.

**Figure 1:**
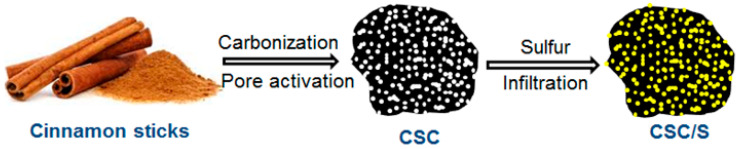
Schematic illustration of the preparation of porous carbon from cinnamon sticks and sulfur infiltration using the melt infiltration method.

In this study, a highly porous carbon with an ultra-high surface area and large pore volume was derived from cinnamon sticks using carbonization and pore activation processes (hereafter referred to as CSC) and utilized as an effective lithium polysulfide reservoir in sulfur cathodes (Figure 1). Cinnamon is a spice, and categorized under the species “Cinnamomum”. Cinnamon is the inner bark of the tree species, and the barks are general byproduct from furniture industries and sawmills. Wooden barks are an inexpensive and abundantly available biomass precursor that could enable high energy and green energy storage devices. However, the bio-mass precursors should be properly engineered to obtain a porous carbon with favorable properties. Herein, the influence of different textural and structural properties of porous carbon on Li–S battery performance is briefly studied. The porous carbon with hierarchical pore structures facilitated faster ionic transport and aided efficient electrolyte absorption, while the large pore walls acted as effective traps for the soluble intermediate lithium polysulfides. The current research studies the possibilities of utilizing green and sustainable materials for next-generation energy storage systems.

## 2. Materials and Methods

High-quality cinnamon sticks were purchased from a local market and were used as a sustainable bio-precursor for the preparation of porous carbon. The cinnamon sticks were broken into small pieces and washed well with distilled water and ethanol several times. The pieces were then vacuum-dried at 120 °C overnight and carbonized at 300 °C. Chemical activation using KOH was adapted to generate the pores, and the ratio of carbon and KOH was controlled to be 1:5. After activation in a tube furnace at 700 °C for 2 h in Ar atmosphere, the product was washed with 0.1 M HCl solution, followed by thorough rinsing with water and ethanol several times. The material was then dried and used for further characterization. A conventional melt impregnation technique was used to infiltrate the elemental sulfur into the pores. The weight ratio of carbon to sulfur was controlled to be 70:30, and these materials were mixed well and infiltrated at 155 °C for 12 h under vacuum. For comparison, CSC activated by different chemical agents—ZnCl_2_ and H_3_PO_4_ were also prepared, and the detailed procedure and characterization of the porous carbon is given in our previous publication [36]. The elemental sulfur was impregnated into CSC—ZnCl_2_ and CSC—H_3_PO_4_ by the same technique as above, and the weight ratio of carbon to sulfur was also controlled to be 70:30.

### 2.1. Electrode Fabrication and Cell Assembly

The pristine sulfur cathode was prepared by casting a slurry of sulfur/super P carbon/poly(vinylidene fluoride) (PVdF) mixed in a ratio of 49/36/15 wt.% in N-methyl-2-pyrrolidone (NMP) onto an aluminum foil. In the case of the CSC/S cathode, the slurry was prepared in a ratio of 70:15:15 (CSC/S:super P:PVdF). The cast electrode was dried in a vacuum oven at 50 °C for 15 h, and the mass loading of sulfur was controlled to be about ~3 mg cm^−2^. The 2032-type coin cells were assembled with the prepared cathode. A 100-µm-thick lithium metal was used as an anode and a polyethylene separator (Asahi ND 420). 1 M lithium bis(trifluoromethanesulfonyl)imide (LiN(SO_2_CF_3_)_2_, LiTFSI) in a mixed solvent of 1,3-dioxalane (DOL) and 1,2-dimethoxyethane (DME) (1:1 by volume) containing 0.4 mol% of lithium nitrate was used as the electrolyte. All the cells were assembled in a dry box with an Ar atmosphere.

### 2.2. Characterization and Measurements

The morphologies of the CSC and CSC/S samples were characterized using a SEM (JEOL JSM 6701F) equipped with EDS spectroscopy and HRTEM (JEOL, JEM 2100F) measurements. XRD (Rigaku D/MAX 2500 diffractometer) was used to characterize the crystalline structure of CSC and CSC/S. XPS (VG multilab ESCA System, 220i) was used to confirm carbonization and to characterize the surface functional groups. Nitrogen adsorption/desorption isotherms were recorded using an Autosorb-IQ MP (Quantachrome Inc., Boynton Beach, FL, USA) apparatus at 77 K. The powder samples were degassed at 100 °C for 3 h prior to the measurements. The amount of sulfur incorporated into the pores of CSC were determined using TGA measurements using a Mettler Toledo SDTQ600 instrument with a heating rate of 10 °C min^−1^ in nitrogen atmosphere from room temperature to 500 °C. Raman spectroscopy was carried out using a Dongwoo Optron, MonoRa 780i spectrometer. 2032-type coin cells were used for the AC impedance measurements using a Zahner Electrik IM6 impedance analyzer in the frequency range of 20 mHz to 100 kHz with an amplitude of 10 mV. CV was measured using a Zahner Electrik IM6 potentiostat in the potential range of 1.8 to 3.0 V at a scan rate of 50 µV s^−1^. Finally, the charge–discharge cycling tests and rate performance measurements were carried out at various current rates in the potential range of 1.8–3.0 V at 25 °C using a battery cycler (WBCS 3000, WonA Tech Co., Ltd., Seoul, Korea). All the potentials reported in the electrochemical measurements are with respect to Li/Li^+^.

## 3. Results and Discussion

The synthesis of hierarchically porous carbon from cinnamon sticks using a two-step process (carbonization and pore-activation) is schematically illustrated in Figure 1. This involved carbonization of the ground cinnamon stick powder at 300 °C, followed by pore activation at 700 °C using KOH powder. Furthermore, sulfur was infiltrated into the pores of CSC using a melt infiltration method (hereafter referred to as CSC/S).

**Figure 2:**
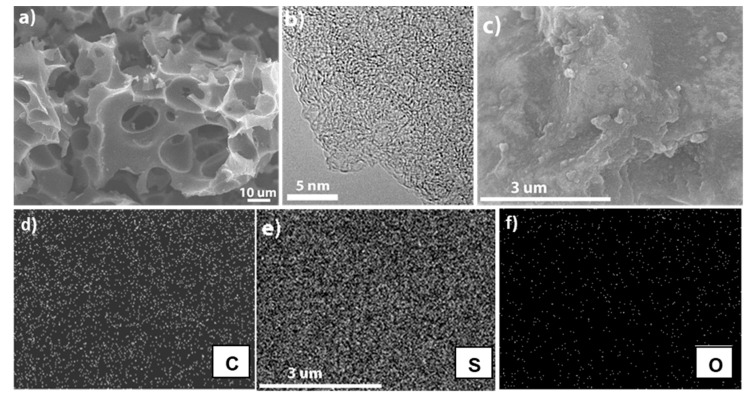
(**a**) SEM, (**b**) HR-TEM images of CSC showing the presence of hierarchical pore structures with macropores, mesopores and micropores. (**c**) SEM image and its corresponding EDS spectrum; (**d**) carbon, (**e**) sulfur and (**f**) oxygen SEM EDS elemental maps for the CSC/S.

The morphology of the CSC was characterized using scanning electron microscopy (SEM) and high-resolution transmission electron microscopy (HR-TEM), as shown in Figure 2. The SEM and TEM images in Figure 2a,b show the presence of macroporous and mesoporous/microporous structures, respectively. This means that CSC possesses hierarchical pore structures that are well distributed and well-connected in a 3-D architecture. As mentioned earlier, this hybrid structure consisting of micro-, meso- and macropores can accommodate a large amount of sulfur and improve lithium ion kinetics by providing easy access to the electrolyte. In addition, the large volume expansion of sulfur during lithiation can be easily accommodated by this hybrid structure, and the strong carbon framework can maintain the electrode architecture without any deterioration, resulting in an enhanced stability. Moreover, the well-connected carbon framework can conduct electrons easily and provide shorter routes for them to access the sulfur electrode, thereby synergistically enhancing the cell performance at higher current rates. Furthermore, the elemental mappings of CSC/S (Figure 2c–f) using SEM energy dispersive spectroscopy (EDS) show the uniform presence of sulfur throughout the sample along with a uniform distribution of carbon and oxygen elements. The amount of infiltrated sulfur was estimated to be 68.7 wt.%, as determined from SEM EDS analysis (Figure S1), indicating a high sulfur loading in the CSC/S sample.

**Figure 3:**
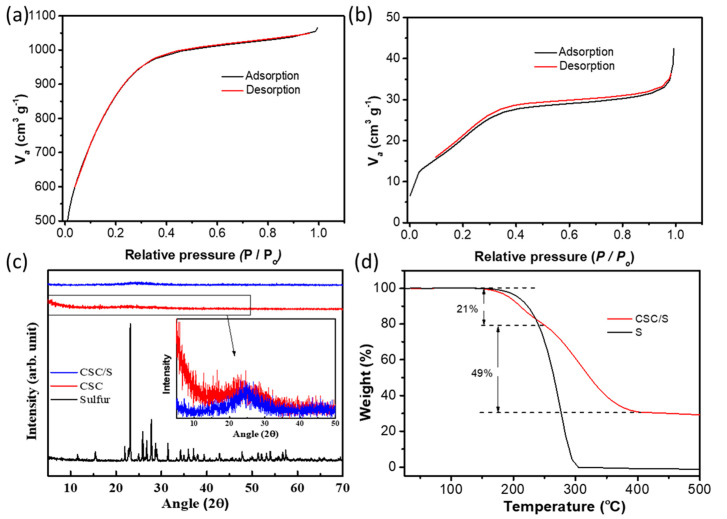
Nitrogen adsorption/desorption isotherms of (**a**) CSC and (**b**) CSC/S; (**c**) XRD patterns of pristine sulfur, CSC and CSC/S, and (**d**) TGA curves of pristine sulfur and CSC/S samples measured in a nitrogen atmosphere.

The textural properties of CSC and CSC/S were analyzed using nitrogen adsorption–desorption isotherms, and the curves are given in Figure 3a,b respectively. The corresponding pore size distribution of CSC and CSC/S was given in Figure S2. The nitrogen adsorption/desorption curves of CSC exhibit a combination of IUPAC Type I and Type IV isotherms with a steep initial region at a low relative pressure, which indicated the presence of mesopores. In addition, the presence of wide knees in the isotherm along with a slight hysteresis loop at a high relative pressure demonstrated the presence of a considerable amount of small mesopores. The Brunauer–Emmet-Teller (BET) surface area was calculated to be 3180 m^2^ g^−1^, with a high pore volume of 1.64 cm^3^ g^−1^, among which 23% were mesopores, rendering it a suitable candidate for embedding sulfur active material. The obtained surface area and pore volume values are one of the highest ever reported for carbon derived from various bio-derived precursors. The mean pore diameter was found to be 2.06 nm. Such a high pore structure was attributed to the activation process at a considerably lower temperature with a high amount of KOH activation agent. The mechanism of pore formation is described as follows. During chemical activation, metallic potassium was intercalated into the carbon framework, and the pores were formed when this metallic potassium was removed by washing. CO_2_ and H_2_O formed during the reaction also improved the porosity of the carbon by acting as a physical activation agent. Moreover, the minerals present in the precursor also played a major role in pore formation by acting as an in-situ activation agent. On the other hand, the adsorption/desorption isotherms of CSC/S showed highly reduced adsorption volumes, and the surface area was determined to be ~100 m^2^ g^−1^, with 22% of this representing mesopores. This highly reduced surface area and pore volume (0.06 cm^3^ g^−1^) in the CSC/S sample indicates that sulfur was impregnated predominantly into the porous structures. The large number of micropores can easily accommodate the elemental sulfur during the melt impregnation process. Moreover, this result demonstrates that not all pores were occupied by sulfur atoms during impregnation, and the unoccupied pores were able to accommodate volume expansion during lithiation and further served as polysulfide reservoirs.

The XRD pattern of CSC in Figure 3c shows a highly amorphous nature, depicting the highly disordered structure of carbon after the activation step. The highly amorphous nature indicates the lack of stacking periodicity of graphene planes in CSC. During chemical activation, the oxygen functionalities get introduced into carbon domains and break down the aligned structural domains to create a large number of slit-like pores [37,38] The high intensity of other amorphous peaks at lower angles confirmed the presence of a high density of pores, and the low peak intensity at 2θ = ~44° indicated that the carbon had a disordered turbostratic nature [39,40]. The mean interlayer spacing (d_002_) was calculated to be 0.391 nm, which was higher than the interlayer spacing of graphite (0.335 nm), confirming the broadening and rearrangement of the structure after activation. The *R*-factor (a representation of the percentage of single-layer graphene in the carbon) was calculated from the ratio between the (002) plane and its background and given in Table 1. In the case of pristine sulfur, highly crystalline peaks corresponding to orthorhombic sulfur (JCPDS No. 08-0247) were observed. However, sulfur impregnation into the porous carbon resulted in a loss of crystallinity of sulfur, which was attributed to the confinement of sulfur into the mesopores of CSC and its strong interaction with the pore walls, which hindered long-range ordering. The amount of sulfur infiltrated into the pores of CSC was confirmed using thermogravimetric analysis (TGA), and thermograms of pristine sulfur and CSC/S are given in Figure 3d. Pristine sulfur completely evaporated in a single step below 300 °C, whereas sulfur loss occurred in two stages in the CSC/S sample at a relatively higher temperature, indicating a stronger affinity with carbon. The first weight loss occurred in the region of 170–250 °C, and the second stage of weight loss occurred in the temperature range of 250–360 °C; these were attributed to the loss of sulfur from mesopores and micropores, respectively [41]. Moreover, 31 wt.% of the sample remained at 400 °C in the CSC/S sample, which corresponded to the carbon content in the composite. This value matches well with the results obtained from SEM EDS analysis. Such a high sulfur loading of 70 wt.% was possible due to the high surface area and large pore volume of CSC.

The successful carbonization of cinnamon sticks was established using X-ray photoelectron spectroscopy (XPS), and the survey and high-resolution C 1s spectra of CSC are given in Figure S3a,b, respectively. The survey spectrum shows the presence of carbon and oxygen with a C/O ratio of 5.7, indicating the presence of functional groups on the surface of the carbon. The presence of such a high amount of surface functional groups was attributed to the relatively lower temperature used in the pore activation step. Besides the porosity and other structural parameters, functional groups over the carbon surface also play a crucial role in the performance of sulfur cathodes in Li–S cells. For example, the presence of functional groups helped enhance the wettability of the electrolyte and enhanced the ability of the carbon to bind lithium polysulfides. To establish the type of functional groups present on the surface, the high-resolution C 1s spectrum was deconvoluted into three peaks centered at 284.6, 285.9 and 289.1 eV, respectively. The peak at 284.6 eV confirmed the presence of sp^2^-hybridized carbon. The peak at 285.9 eV was assigned to the hydroxyl groups, and the other broad peak at a higher binding energy was attributed to the carbonyl and carboxyl groups. The presence of characteristic D- and G-bands at 1354 and 1597 cm^−1^, respectively, in the Raman spectrum of CSC (Figure S4) further confirmed the successful carbonization of cinnamon sticks [42]. The D-band indicated the presence of disordered carbon, and the G-band arose from the E_2g_ vibration mode of sp^2^-hybridized graphitic carbon. In addition, the I_D_/I_G_ ratio, which indicates the extent of defects, was determined to be 0.98, which is comparable with other works from the literature.

**Figure 4:**
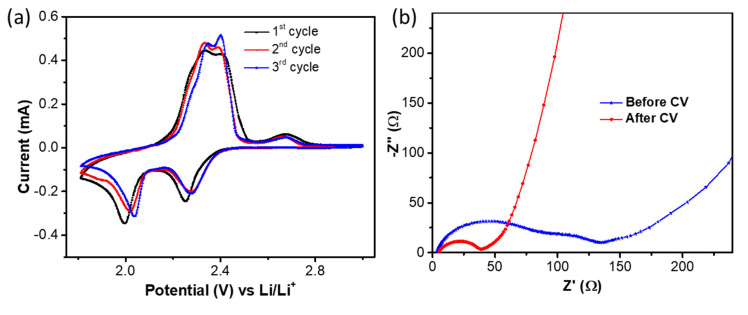
(**a**) Cyclic voltammograms and (**b**) AC impedance spectra of the CSC/S electrode.

The cyclic voltammogram (CV) of the CSC/S electrode in the potential range of 1.8 to 3.0 V is given in Figure 4a. Two well-defined cathodic peaks at ~2.2 and 2.0 V and an overlapping anodic peak at 2.3 V were observed, which are typical for sulfur electrodes. The elemental sulfur was converted into long-chain lithium polysulfides (Li_2_S_n_, 4 < n ≤ 8) at ~2.2 V, followed by the formation of short-chain lithium polysulfides (Li_2_S_2_/Li_2_S) at 2.0 V during the cathodic process. During the reverse cycle, an overlapped anodic peak was observed at ~2.3 V, which was assigned to the re-formation of cyclic sulfur. The cathodic peaks were positively shifted to a higher potential in the subsequent cycles, indicating the decreased overpotential. This could be attributed to the favorable structural rearrangement in the CSC/S electrode induced by the volume changes during cycling. This phenomenon was further substantiated by the decrease in the overall impedance of the cell upon cycling, as observed in the AC impedance spectra before and after three cycles of CV measurements (Figure 4b). The electrolyte resistance and charge transfer resistance of CSC/S before and after CV are given in Table S1. The reduction in the charge transfer resistance is attributed to the electrode activation process in Li–S cells. Upon cycling, the electrolyte gets infiltrated into the bulk of the electrode and increases the electrode–electrolyte contact to increase the lithium kinetics. A slight increase in electrolyte resistance reflects the development of a slight surface resistance layer after the cycling process [43]. A mechanistic and system-level modeling of Li–S batteries can provide a clear picture of the interface of the sulfur electrode and electrolyte [44].

**Figure 5:**
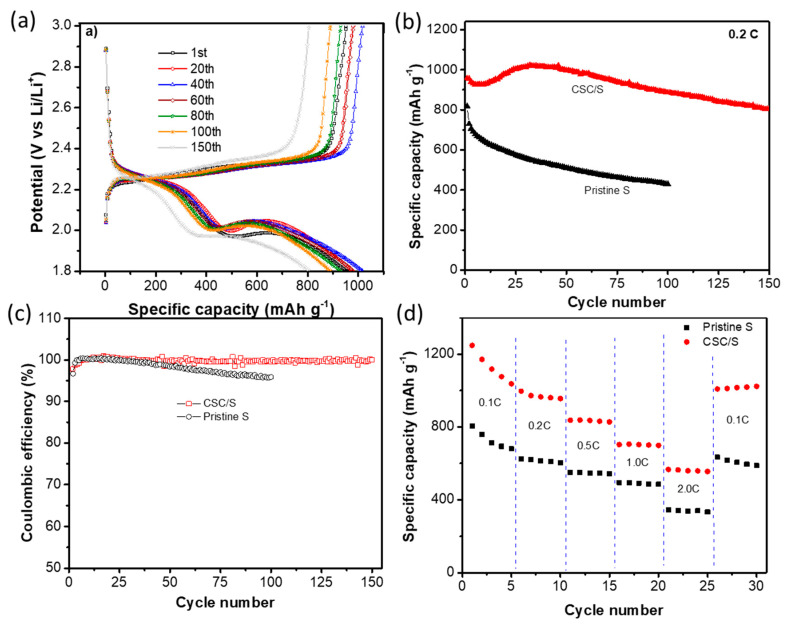
Electrochemical characterization of CSC/S as a cathode in the Li–S cells: (**a**) Charge/discharge voltage profiles at various cycles at a rate of 0.2C, the corresponding (**b**) cycling performance and (**c**) coulombic efficiency plots, and (**d**) the rate performance of the CSC/S electrode at various incremental C rates.

Furthermore, the cycling performance of the Li–S cell with a CSC/S cathode was evaluated at a 0.2C rate (1C = 1675 mAh g^−1^), and the charge–discharge voltage profiles at different cycles are given in Figure 5a. As observed from the CV measurements, the discharge curves exhibited two plateaus at 2.2 V and 2.0 V, and the charge curves showed a single overlapped plateau. The upper discharge plateau remained relatively stable up to 100 cycles and started to decline upon further cycling. The relatively stable upper discharge plateau indicated that the loss of active material through the diffusion of soluble lithium polysulfides was greatly reduced [45]. This indicated that the unoccupied micro-/mesopores acted as lithium polysulfide reservoirs and prevented the migration of soluble polysulfides to the anode side. In addition, the overpotential decreased during the initial cycles in comparison to the first cycle and then started increasing after 60 cycles. As explained above, the decreased overpotential in the initial cycles was attributed to the favorable structural rearrangement of sulfur within the pores and activation of electrochemically inactive sulfur through enhanced electrolyte wetting. However, upon continuous cycling, the discharge products accumulated on the pore walls, thereby resulting in an increase in the overpotential. The cycling performance of the Li–S cell assembled with the CSC/S electrode was compared to the cell with a pristine sulfur cathode in Figure 5b. The cell with the CSC/S cathode showed a slight drop in capacity in the initial few cycles, and the capacity increased upon further cycling. The increase in capacity after a few cycles was attributed to the activation of sulfur facilitated through structural rearrangement caused by volume changes upon cycling. A maximum specific discharge capacity of 1020 mAh g^−1^ was observed with a capacity fading rate of 0.10% per cycle. In contrast, the cell with pristine sulfur showed a lower initial discharge capacity, and the capacity fading was more pronounced with a fading rate of 0.47% per cycle. The capacity fading of Li–S cells with cycling was mainly associated with the shuttle reaction of long-chain lithium polysulfides [46]. The enhanced cycling stability of the cell with the CSC/S electrode demonstrated that the pore structures with a high surface area and large pore volume of CSC acted as reservoirs for lithium polysulfides. Moreover, the cell with the CSC/S cathode showed a stable coulombic efficiency above 99% (Figure 5c), which was far superior to the coulombic efficiency of the cell with the pristine sulfur cathode. The long-term cyclic stability of CSC activated by ZnCl_2_ and H_3_PO_4_ was also studied for the Li–S battery (Figure S5). It can observed that the other two porous carbons show a more rapid and quick capacity fading than CSC–KOH. This is attributed to a lower surface area and pore volume of CSC–ZnCl_2_ and CSC–H_3_PO_4_ carbons, which could not efficiently infiltrate elemental sulfur and trap polysulfides during the cycling process. This indicates the need for a larger pore volume in porous carbons that function both as a highly conductive network for sulfur and as a polysulfide reservoir for polysulfides during cycling. To demonstrate the improved kinetics of Li–S cells, the rate capability was evaluated at various current rates from 0.1C to 2C every five cycles at each current, and the results are given in Figure 5d. The cell with a CSC/S cathode exhibited a higher specific capacity at all the tested current rates as compared to the pristine sulfur cathode. The cell with a CSC/S electrode delivered a discharge capacity of 536 mAh g^−1^ at a 2C rate. Table S2 compares the electrochemical performance of CSC/C with other bio-mass-derived carbon studied for Li–S batteries. It can be noted that CSC/C delivers a high-capacity, improved cyclic stability and a high rate capability, even with 70 wt.% sulfur, outperforming the previous reports. The large pore volume of CSC greatly helps to accommodate a large sulfur content. In contrast, the pristine electrode delivered a discharge capacity of 362 mAh g^−1^ at the same current rate. Although both the electrodes contained a similar amount of sulfur and conductive carbon, the CSC/S electrode showed a superior rate performance. This was attributed to the presence of hierarchical and well-connected pore structures, which aided in a faster lithium ion diffusion as well as an enhanced electronic conductivity. The enhanced ionic and electronic conduction resulted in faster reaction kinetics.

## 4. Conclusions

A hierarchically porous carbon with a high surface area and large pore volume was synthesized from a sustainable bio-mass, and its utility in sulfur cathodes as a polysulfide reservoir was successfully demonstrated. The presence of hierarchical and well-connected pore structures enhanced the electronic conductivity and ionic diffusion, resulting in faster reaction kinetics. In addition, the polysulfides were effectively trapped by the micro/mesoporous adsorption sites, resulting in an enhanced cycling performance. This simple strategy of using bio-derived porous carbon to enhance the cycling performance of Li–S cells can pave the way for environmentally friendly alternatives for high energy applications.

## Figures and Tables

**Table 1:** Structural parameters, and pore parameters of CSC.

d(002) nm	R	S_BET_ (m^2^ g^−1^)	V_t_ (cm^3^ g^−1^)	Micropores (%)	Mesopores (%)
0.391	2.52	3180	1.64	77	23

## Supplementary Materials

The following are available online at https://www.mdpi.com/2079-4991/10/6/1220/s1, Figure S1: SEM EDS spectrum of CSC/S sample. Figure S2: Pore size distribution plots of (**a**) CSC and (**b**) CSC/S. Figure S3: (**a**) XPS Survey of CSC, and (**b**) De-convoluted high-resolution C 1s XPS spectra of CSC. Figure S4: Raman spectrum of CSC sample showing the presence of G- and D-bands. Figure S5: Comparison of cyclic stability (0.2C) of CSC/S activated with different chemical agents. Table S1: Electrolyte solution resistance, and charge transfer resistance of CSC/C before and after CV. Table S2: Table comparing the textural properties of CDC, and electrochemical performance of CSC/S with other bio-mass carbon.
