# Supplementary material for: Cinnamon-Derived Hierarchically Porous Carbon as an Effective Lithium Polysulfide Reservoir in Lithium–Sulfur Batteries
Source: Nanomaterials (Basel). 2020 Jun 22;10(6):1220. doi: 10.3390/nano10061220 (PMC7353079; doi:10.3390/nano10061220)
Supplement: Supplementary file 1 [file nanomaterials-10-01220-s001.pdf]

Supporting Information

Biomass-derived Hierarchically Porous Carbon as an Effective Lithium Polysulfide Reservoir in Lithium-Sulfur Batteries

Ranjith Thangavel [1, 2,†] Aravindaraj G Kannan [3,†] Rubha Ponraj [3], Karthikeyan Kaliyappan[4], Won-Sub Yoon [2], Dong-Won Kim [3,*] and Yun-Sung Lee [1,*]

[1] School of Chemical Engineering, Chonnam National University, Gwang-ju, 61186, Republic of Korea
[2] Department of Energy Science, Sungkyunkwan University, Suwon, 16419, Republic of Korea
[3] Department of Chemical Engineering, Hanyang University, Seoul, 04763, Republic of Korea
[4] Department of Chemical Engineering, University of Waterloo, Waterloo, Ontario, N2L 3G1, Canada
* Correspondence: dongwonkim@hanyang.ac.kr (D.–W. Kim); leeys@chonnam.ac.kr (Y.-S.Lee)
† Equal contribution

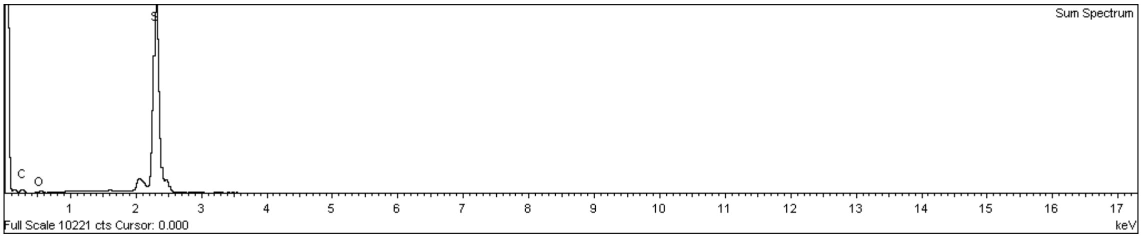

Figure S1. SEM EDS spectrum of CSC/S sample. The amounts of carbon, oxygen and sulfur were determined to 29.5, 1.8 and 68.7 wt.%, respectively.

Figure S2. Pore size distribution plots of (a) CSC and (b) CSC/S.

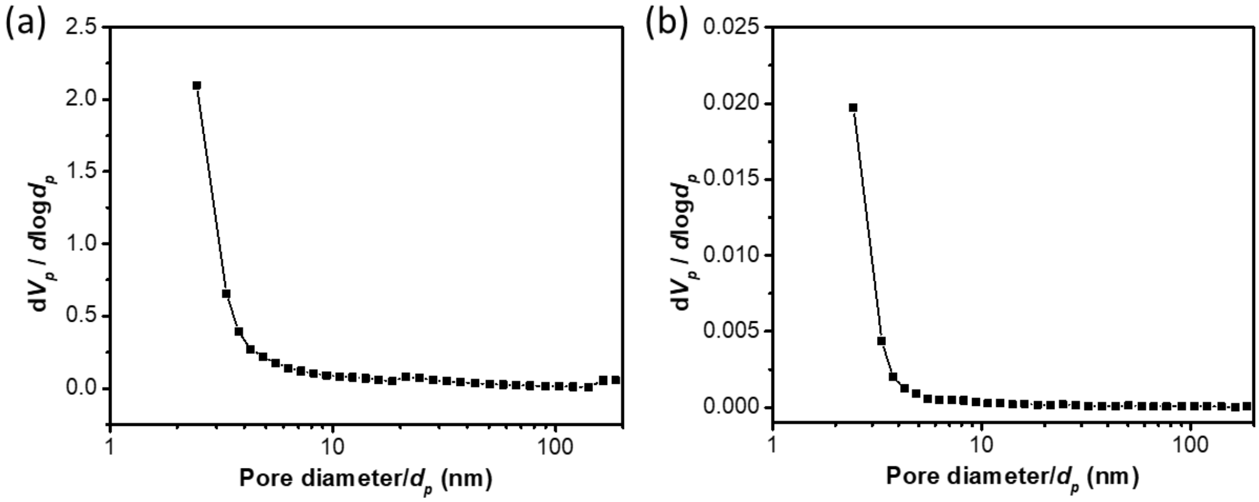

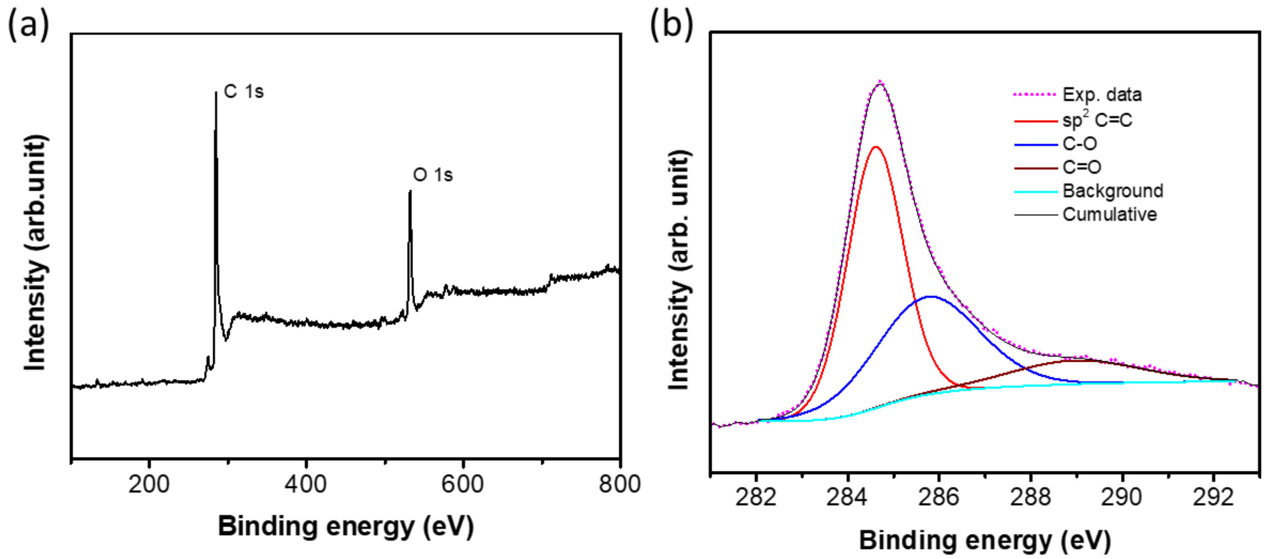

Figure S3. (a) XPS Survey of CSC, and (b) De-convoluted high-resolution C 1s XPS spectra of CSC

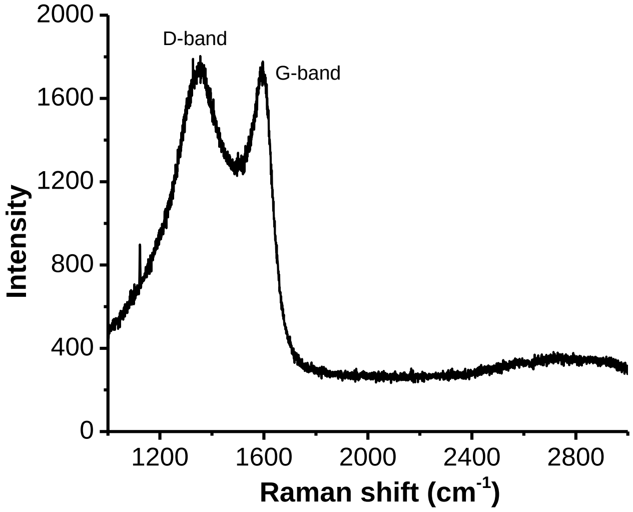

Figure S4. Raman spectrum of CSC sample showing the presence of G- and D-bands.

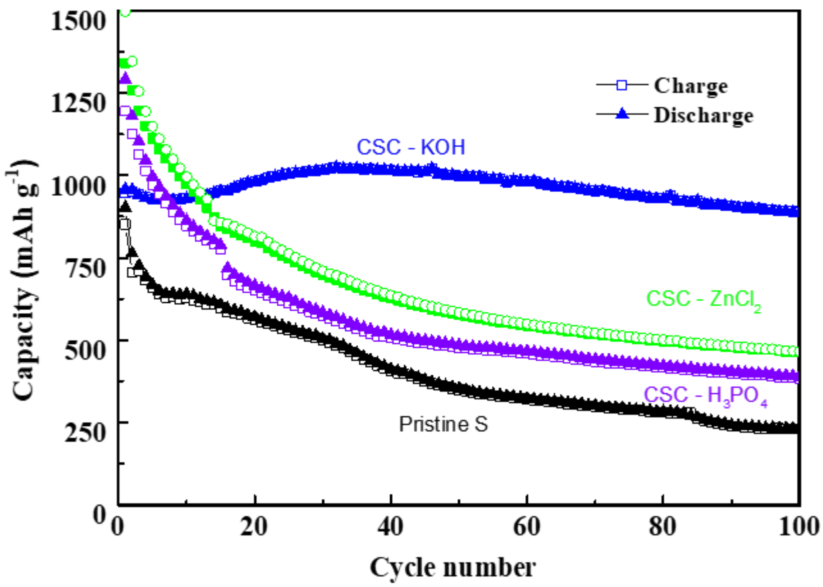

Figure S5. Comparison of cyclic stability (0.2C) of CSC/S activated with different chemical agents.

	Solution resistance (R_s) Ω	Charge transfer resistance (R_{ct}) Ω
Before CV	3.3	130.96
After CV	4.5	34.27

Table S1. Electrolyte solution resistance, and charge transfer resistance of CSC/C before and after CV

	Activation agent	Surface area ($m^2\ g^{-1}$)	Pore volume ($cm\ g^{-1}$)	Discharge capacity ($mAh\ g^{-1}$)	Stability	Sulfur content (wt.%)
Cinnamon (This work)	KOH	3180	1.64	1248	85% - 150 cycles (0.2 C)	70
Apricot shell[1]	KOH	2269	1.05	1277	55.6% - 200 cycles (0.2 C)	53.5
Pomelo peel[2]	KOH	1533	0.84	1258	59.6% - 100 cycles (0.2 C)	60
Walnut shell[3]	KOH	2318	1.137	1350	67.4% - 100 cycles (0.1 C)	48.8
Bamboo[4]	KOH	776	0.33	907	66.7% - 300 cycles (1 C)	70
Goat Hair[5]	H_3PO_4	535.4	0.39	1185	41.2% - 300 cycles (0.2 C)	54.33
Cotton[6]	KOH, Urea	1286	1.15	1017	74.7% - 200 cycles (0.2 C)	68
Silk cocoons[7]	KOH	3243	2.1	1443	55.12% - 80 cycles (0.5 C)	48

Table S2. Table comparing the textural properties of CDC, and electrochemical performance of CSC/S with other bio-mass carbon.[1-7]

1. Yang, K.; Gao, Q.; Tan, Y.; Tian, W.; Zhu, L.; Yang, C. Microporous carbon derived from Apricot shell as cathode material for lithium–sulfur battery. *Microporous and Mesoporous Materials* **2015**, *204*, 235-241.

2. Zhang, J.; Xiang, J.; Dong, Z.; Liu, Y.; Wu, Y.; Xu, C.; Du, G. Biomass derived activated carbon with 3D connected architecture for rechargeable lithium−sulfur batteries. *Electrochim. Acta* **2014**, *116*, 146-151.

3. Liu, J.; Liu, B.; Wang, C.; Huang, Z.; Hu, L.; Ke, X.; Liu, L.; Shi, Z.; Guo, Z. Walnut shell – Derived activated carbon: Synthesis and its application in the sulfur cathode for lithium–sulfur batteries. *J. Alloys Compd.* **2017**, *718*, 373-378.

4. Gu, X.; Lai, C.; Liu, F.; Yang, W.; Hou, Y.; Zhang, S. A conductive interwoven bamboo carbon fiber membrane for Li–S batteries. *J. Mater. Chem. A* **2015**, *3*, 9502-9509.

5. Ren, J.; Zhou, Y.; Wu, H.; Xie, F.; Xu, C.; Lin, D. Sulfur-encapsulated in heteroatom-doped hierarchical porous carbon derived from goat hair for high performance lithium–sulfur batteries. *Journal of Energy Chemistry* **2019**, *30*, 121-131.

6. Wang, H.; Chen, Z.; Liu, H.K.; Guo, Z. A facile synthesis approach to micro–macroporous carbon from cotton and its application in the lithium–sulfur battery. *RSC Advances* **2014**, *4*, 65074-65080.

7. Zhang, B.; Xiao, M.; Wang, S.; Han, D.; Song, S.; Chen, G.; Meng, Y. Novel Hierarchically Porous Carbon Materials Obtained from Natural Biopolymer as Host Matrixes for Lithium–Sulfur Battery Applications. *ACS Appl.Mater. Interfaces* **2014**, *6*, 13174-13182.
